# Rewilding soil-disturbing vertebrates to rehabilitate degraded landscapes: benefits and risks

**DOI:** 10.1098/rsbl.2022.0544

**Published:** 2023-04-05

**Authors:** David J. Eldridge, Santiago Soliveres

**Affiliations:** ^1^ Centre for Ecosystem Science, School of Biological, Earth and Environmental Sciences, University of New South Wales, Sydney, New South Wales 2052, Australia; ^2^ Department of Ecology, Universidad de Alicante, Carretera de San Vicente del Raspeig s/n, C.P. 03690 San Vicente del Raspeig, Alicante, Spain; ^3^ Instituto Multidisciplinar para el Estudio del Medio ‘Ramón Margalef’, Universidad de Alicante, Carretera de San Vicente del Raspeig s/n, C.P. 03690 San Vicente del Raspeig, Alicante, Spain

**Keywords:** soil function, restoration, biopedturbation, soil disturbance, rewilding

## Abstract

Soil-disturbing animals are common globally and play important roles in creating and maintaining healthy functional soils and landscapes. Yet many of these animals are threatened or locally extinct due to habitat loss, predation by non-native animals or poaching and poisoning. Some reintroduction and rewilding programmes have as their core aims to increase animal populations and reinstate processes that have been lost due to their extirpation. Here we use a meta-analytical approach to review the effects of soil-disturbing vertebrates on ecosystem processes, and advance the argument that they can be used to rehabilitate degraded ecosystems by altering mainly composition and function, but with fewer positive effects on structure. We describe four examples where the loss or reintroduction of soil-disturbing vertebrates leads to ecosystem state changes and highlight the role of spatial scale, covarying management changes, and species co-occurrence in modulating their effects. We discuss the advantages and disadvantages of using soil-disturbing vertebrates over mechanized engineering approaches such as pitting and furrowing, considering some advantages to include more self-sustainable and heterogeneous disturbances, creation of new habitats and added recreational values. Finally, we identify key knowledge gaps in our understanding of the use of soil-disturbing vertebrates for rehabilitating degraded ecosystems.

## 1. Introduction

When animals disturb the soil to forage, rest, nest or escape from predators, they create two types of unique structures or microsites: (i) degradational (e.g. pit, hole and depression) or (ii) aggradational (e.g. accumulating soil or ejecta from the hole). These two types of structures have marked effects on soil and ecological properties and processes, including changes in vegetation and soil biota composition, nutrient accumulation rates or water infiltration capacity (electronic supplementary material, figure S1). Many of these soil-disturbing vertebrates (e.g. small rodents, lizards and birds) are severely threatened by exotic predators, habitat loss, hunting and even poisoning. Populations of many species of soil-disturbing vertebrates have abruptly declined over the past few decades [1], although there are also a few ‘new arrivals’ (e.g. rabbits and pigs outside Europe [2]), or native recolonizations, due to recent conservation efforts (e.g. bilbies in Australia). Given the importance of their ecosystem effects, it is not unexpected that the loss and compositional changes of soil-disturbing vertebrates should lead to substantial changes in the functions that they mediate. Conversely, bringing their original populations back to what they were in the past (i.e. rewilding, [3]) has the potential to restore soil and vegetation processes that can have positive feedback effects on biodiversity and productivity of many ecosystems globally. However, evidence for rewilding with soil-disturbing vertebrates is still limited, and there are potential pitfalls to be considered when reintroducing these animals.

Here we produce an overview of the current extent and composition of soil-disturbing vertebrates globally, review existing literature on their impacts on a range of ecological processes (composition, structure and function) and describe four examples that illustrate how their activity and extirpation can lead to ecosystem state changes. Finally, we discuss where and how the reintroduction of these animals can help to restore biodiversity and ecosystem processes more cheaply and sustainably than engineering-based restoration techniques such as furrowing and pitting.

## 2. Soil disturbance enhances biodiversity and productivity

Animal foraging mobilizes sediment that is either trapped in the mound (the ejecta sediment) or transported through the landscape by wind and water (electronic supplementary material, figure S1). Globally, contemporary rates of soil formation are orders of magnitude less than rates of soil loss, so soil disturbance by animals has the potential to lead to the formation of novel soil horizons. Annual rates of soil movement vary widely among taxa from 0.1 t ha^−1^ for solitary marsupials [4] to 22 t ha^−1^ for colonial pocket gophers [5] and more than 350 t ha^−1^ for feral pigs [2].

Pits and depressions moderate hydrological processes by retarding runoff [6,7] and therefore enhancing retention of water and sediment. Pits are also more porous and trap moisture in their lower layers [8,9], even during extended dry periods [10]. Because foraging pits catch sediment and organic material (seed and litter, electronic supplementary material, figure S1), they gradually infill until they no longer act as resource traps [11]. Capture and infilling result in the trapping of litter beneath the surface where it comes into contact with microbes and microarthropods. Greater decomposition within the pits [9] enhances soil carbon and nitrogen pools [10,12,13], and depending on pit age [7,11,14], alters soil microbial communities [14,15]. Litter capture also accelerates phosphorus turnover [7]. Foraging pits and mounds also result from other animal disturbances such as those constructed when animals create shelter (e.g. hip holes and resting forms [16,17]).

Overall, greater soil moisture and fertility within pits create favourable microsites for seed germination and establishment [11,13,18–20]. This often leads to different plant composition from that on undisturbed ground [21]. Importantly, these positive effects prevail and may even increase under unproductive conditions [7], providing an opportunity to use animal activity to rehabilitate soils in water-limited (dryland) environments. Some of these positive effects may be dampened by the trophic effects of soil-disturbing vertebrates, which are commonly herbivores or omnivores. However, we know little about densities that optimize the ecosystem value of soil-disturbing animals, and this requires studies of ecosystem functions under different densities, often using experimental approaches [11] and predictive models (table 1). Though disturbances may increase plant diversity, herbivory could result in compensatory reductions in biomass [19,22] depending on the levels of disturbance [23–24]. Indeed, excessive disturbance (e.g. those created by feral pigs [24]) can lead to reduced vegetation cover.

**Table 1.** Open research questions on the ecological impacts of soil-disturbing vertebrates. References supporting these statements are available in the main text.

topic	knowledge gaps	approach recommended
carrying capacity for soil-disturbing animals	we currently lack information on optimal densities of soil-disturbing animals in order to maximize their ecological benefits across different ecosystems	measure ecosystem functions (e.g. runoff, soil nutrients, plant composition and structure) at different sites under contrasting densities of soil-disturbing animal
		use predictive models to establish optimal soil movement rates for different processes
predict soil-disturbing animal effects	the impacts of soil-disturbing animals may depend on the traits of the animal itself (e.g. body size and metabolic rate), but also on environmental conditions (e.g. soil texture and depth, climate), or the presence of other species (e.g. livestock)	meta-analyses predicting effect size versus animal traits, and biotic and abiotic conditions. These will indicate species and locations where animals are most beneficial to enhance restoration
		experimental or observational evaluation of the effect of soil-disturbing animal richness and composition on ecosystem functioning
functional overlap between soil-disturbing vertebrates	some ecosystems support a diverse array of soil-disturbing vertebrates (figures 1*a* and 2*c*), but whether these effects are complementary or redundant is unknown. Are there diversity-mediated effects of soil vertebrates on ecosystems?	mimic studies analysing biodiversity-functioning relationships with soil-disturbing vertebrates. Differences in site function could be examined in relation to increasing richness of soil-disturbing species (species richness gradient). Consider also their collective abundance and changes in their composition (and functional traits)
home versus away effects	some animals could have overall beneficial effects in their native range, but mostly negative effects outside their range. How many soil-disturbing animals are invasive outside their native ranges, and how do their effects compare between home and away?	home and away comparisons could: (i) improve our understanding of underlying mechanisms behind the ecological impacts of soil-disturbing animals, (ii) identify those ecological processes that are more effective at maintaining sustainable densities of these animals
		compare population dynamics and ecological impacts of the same soil-disturbing species where it is native and exotic. A widely distributed species (e.g. rabbits) could be a suitable model candidate
cost : benefit analysis of mechanical versus ecological soil engineering	soil-disturbing animals can provide additional services and disservices when reintroduced to restore/rehabilitate degraded land. These co-benefits/disbenefits should be considered when assessing cost : benefit ratios to determine which restoration tools are more appropriate under different scenarios	mid- to long-term experimental comparisons of the effects of mechanical and ecological structures (pits, mounds) on ecosystem functions
		comparisons would benefit from incorporating the role of persistence and heterogeneity (ecological > mechanical) in space and time
		incorporate stakeholder perspectives on recreational and cultural values, potential as prey for other species of interest (including hunting) and other associated services
which ‘reference’ ecosystem do we establish?	as with any ecological restoration approach, one needs to establish the reference to which such an approach should aim. In many areas, the abundance and composition of soil-disturbing vertebrates have been severely altered, either through the introduction of predators or new diseases, habitat loss, or poaching for different reasons	consulting ecological archives and natural history descriptions from previous decades and centuries could provide important information on past composition and abundance of soil-disturbing animal communities. These could be used to establish target references

## 3. A global assessment of vertebrate soil disturbance

**Figure 1.**
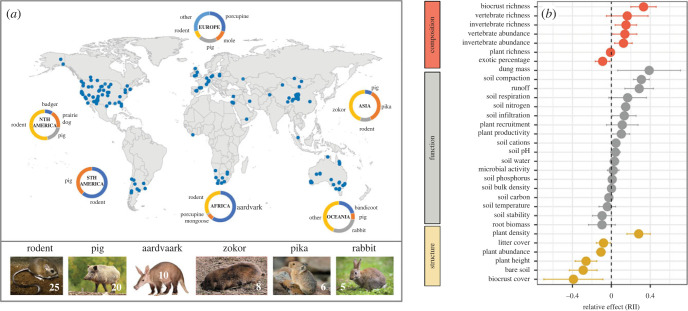
(*a*) Global map of the distribution of data on the relative effects of soil-disturbing animals on average ecosystem response (the average RII value across all attributes, see electronic supplementary material, Methods). Circles show the relative proportion of the four most common broad animal groups, including the ‘other’ group where appropriate, for the six hot spots of soil-disturbing vertebrate research. Lower panel shows the percentage of records for the main soil-disturbing vertebrate groups, i.e. 25% of the 2437 observations were attributed to rodents. (*b*) Mean (±95% CI) of the RII for the 31 attributes arranged by structure, function and composition. Differences between disturbed and control are significant if the CI does not overlap the *x* = 0 line.

To assess the balance between the positive and negative effects of soil-disturbing vertebrates, we updated a global database [7] to include 2437 records of 70 vertebrate species across all continents except Antarctica. We included data published to September 2022. This updated database extends the work of Mallen-Cooper *et al.* [7] by (i) considering the three elements of biodiversity (structure, function and composition) as well as individual attributes and (ii) the effects of different vertebrate species. Our focus here is on the practical application of soil-disturbing animals and how they might be used to rehabilitate degraded systems. The resulting database was used to provide an overview of the composition of these soil-disturbing vertebrates globally, and their overall and group-specific effects on ecosystems (figure 1; electronic supplementary material, figure S2). To do so, we calculated the net effects of soil-disturbing vertebrates using the relative interaction index (RII, [25], electronic supplementary material, Methods). This index ranges from –1 to +1 and positive values indicate a net positive effect due to soil disturbance and *vice versa*. The RII values of all attributes were used separately (figure 1*a,b*), then pooled into three categories (for the 12 most common soil-disturbing vertebrates). These categories provide measures of ecosystem structure (the building blocks of ecosystems, e.g. plant cover and patch size), function (measures that support ecosystem processes such as decomposition and hydrology) and composition (measures of diversity; electronic supplementary material, table S1).

The mean value of structural attributes (electronic supplementary material, table S1) declined significantly under the action of soil-disturbing vertebrates (–0.09 ± 0.04, mean ± 95% CI), mainly due to the negative effects on biocrust and plant abundance, plant height and litter cover, although soil-disturbing animals also enhanced plant densities (figure 1*b*). Overall effects of soil disturbance on composition were generally positive, increasing biocrust richness, invertebrates and vertebrate abundance, and invertebrate richness, but with non-significant effects on plant richness (figure 1*b*). Effects of soil-disturbing vertebrates on function were generally positive (0.06 ± 0.02) but mixed. Soil nitrogen, infiltration and soil water were greater, and likely account for the increased plant production. However, runoff and soil compaction also increased. Evidence to date clearly suggests that rewilding with soil-disturbing vertebrates can greatly benefit biodiversity but that it may come with environmental costs associated with the loss of important attributes of ecosystem structure and functions related to soil compaction, erosion and runoff.

We also found strong species-dependency in the overall effects of soil-disturbing vertebrates on ecosystems. The effects of soil-disturbing vertebrates ranged from predominantly positive effects on ecosystem composition, function and structure in the presence of porcupines, mongooses, prairie dogs and bandicoots, to predominantly negative responses seen in rabbits, pikas or zokors. Rabbits and prairie dogs had generally positive effects on function, but negative effects on structure (electronic supplementary material, figure S2. Although differences in body size do not appear to be driving the functional response of different vertebrates, animal densities, functional traits other than body size or the type of soil disturbance (deep versus superficial) could strongly modulate the sign and intensity of the effects of soil-disturbing vertebrates on ecosystems, which warrants further research (table 1) to aid predictions of the consequences of rewilding different suites of animals that differ markedly across the planet. In addition, positive effects could concentrate in the native range of these species, whereas negative effects could dominate elsewhere, although existing evidence for rabbits and wild pigs are inconclusive in this regard.

## 4. Illustrating how the loss or reintroduction of soil-disturbing animals leads to ecosystem state changes

We now illustrate some of the factors that potentially modulate the direction and strength of the effects of soil-disturbing vertebrates with three relatively well-known examples including density-dependence, complementary effects and the need for additional restoration practices.

**Figure 2.**
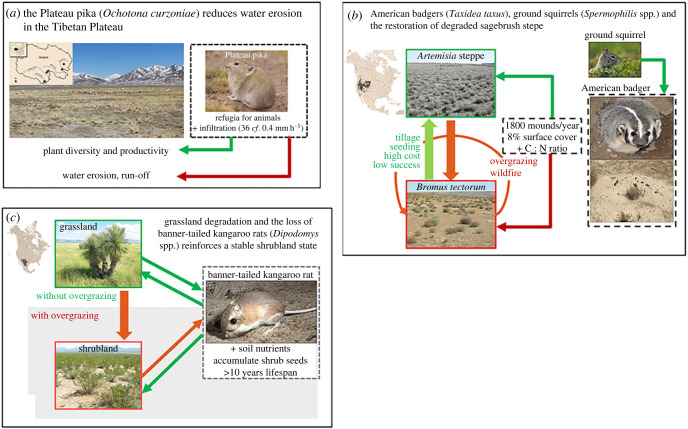
Three examples of showing how the loss or reintroduction of soil-disturbing animals leads to ecosystem state changes: (*a*) Plateau pika on the Tibetan Plateau, China. Infiltration rates in the presence of pika (36 mm h^–1^) or after extirpation (0.4 mm h^–1^); (*b*) American badgers in west-central Idaho USA and (*c*) banner-tailed kangaroo rats in the Chihuahuan Desert, western North America.

The Plateau pika (*Ochotona curzoniae*) is a widely distributed keystone species across the Tibetan plateau and a significant prey item of plateau carnivores. Its burrows are important refugia for birds and reptiles (figure 2*a*) and the mounds play important roles in soil and vegetation dynamics [26]. Yet, pika are poisoned because they are thought to compete with livestock. Pika colonies improve water infiltration [27], so their removal can lead to reduced infiltration and more runoff during monsoon rains. This has potentially large-scale ecosystem impacts on water erosion and siltation of major water storage structures [26]. Pikas are therefore a suitable study case to evaluate optimal animal densities that maximize their benefits preventing erosion and sustaining local predators while avoiding undesirable loss of pastoral value.

The sagebrush (*Artemisia* spp.) steppe is an important biome in North America. Over the past century plant composition has been dramatically altered by overgrazing, invasion by Eurasian exotic grasses (*Bromus tectorum*) and wildfire [28]. Increasing wildfires leads to a reinforcement of annual grasses and therefore more fire. Two soil-disturbing ecosystem engineers [29] are important components of these shrub steppe ecosystems. Ground squirrels (*Spermophilus* spp.) construct small tunnels that enhance water flow into the soil [30]. The American badger (*Taxidea taxus*), in turn, predates upon ground squirrels, excavating their burrows and creating large mounds of excavated soil (figure 2*b*) that occupy up to 8% of the landscape and have soils with a greater C : N ratio (approx. 25–30) than undisturbed soils (approx. 15; [12]). The high C : N ratio makes mounds preferred microsites for native plants (e.g. *Poa secunda*) at the expense of weeds*.* Mounds therefore provide suitable patches from which native plants can re-establish after overgrazing or fire. Hence, there are clear co-benefits of simultaneously rewilding with these two soil-disturbing vertebrates (table 1), and their restoration potential exceeds that of mechanical methods to restore sagebrush, which have largely been unsuccessful.

The banner-tailed kangaroo rat (*Dipodomys spectablis*) is an important soil-disturbing mammal (rodent) from North America's Chihuahuan Desert (figure 2*c*). The nutrient-rich soils associated with its burrows support unique microbial communities and are preferred sites for desert plants [31]. Overgrazing and woody plant increase (shrub encroachment) have led to changes in nutrient levels and sometimes desertification [32]. Digging by kangaroo rats can exacerbate shrub encroachment (figure 2*c*) by penetrating the impermeable carbonate layer near the surface, allowing shrubs to access water. Declines in green grass following encroachment force the rats to abandon their mounds [33], promoting larger and denser plants on the mounds [34]. Thus, although the rats maintain plant diversity in intact grasslands, they also reinforce the shrub-encroached state. Engineering-based solutions to restoring these systems have largely been unsuccessful [35], and the reintroduction of soil-disturbing animals such as the kangaroo rat is critical to reinstate functional soil and ecological processes, but it needs to be applied in tandem with reductions of grazing pressure.

## 5. Rewilding the recovery of degraded soils: potentials and risks

Soil-disturbing vertebrates occur across a wide range of ecosystems globally (figure 1*a*), suggesting that their reintroduction could have widespread future benefits. Reintroductions of soil-disturbing vertebrates have been sometimes intentional and with native animals, such as the case of bettongs (*Bettongia* spp.) and bilbies (*Macrotis lagotis*) in Australia [36], and sometimes either accidental or intentional with exotic animals (e.g. pigs, rabbits), that have replaced processes lost by the extinction of functionally similar species [1]. The extent to which these rewilded species are functionally equivalent to the original vertebrates is unknown. Similarly, it is unknown how vertebrates differ between their native and exotic ranges (e.g. European rabbit). Addressing these knowledge gaps requires comparative studies across different environments (table 1).

Soil-disturbing animals may be cheaper and more effective tools for rehabilitation than mechanical methods. For example, the foraging pits of the short-beaked echidna (*Tachyglossus aculeatus*) have a similar configuration to those constructed by the tyne pitter, a mechanical device used to restore degraded soils worldwide (e.g. [37]), but echidna pits offer additional advantages. They vary markedly in shape, size, placement and distribution, which enhance heterogeneity, and support a broader range of plant and litter types, and therefore microbial communities [14]. Additionally, animal-created disturbances are self-sustaining i.e. are maintained and reactivated over time, and cheaper to construct than mechanical pits. Current costs associated with mechanical treatment (tyne pitting) range from U.S. $18–31 per hectare, for fixed (equipment maintenance) and variable (fuel and labour) costs, though the environmental costs are unknown. Rewilding with soil-disturbing vertebrates can also provide additional co-benefits associated with greater carbon sequestration, social and intrinsic benefits associate with supporting native animals, which could further offset the costs of reintroduction. While some of these added benefits are difficult to evaluate economically, such as the provision of habitat for other species by the echidna, for others such as the Plateau pika (figure 2*a*), reductions in soil erosion at large spatial scales can be valued through the avoided cost of desilting dams.

There are also costs and shortcomings of using soil-disturbing animals, which include (i) the longer time period required to ‘treat’ an area equivalent to one mechanically treated, (ii) suitable habitat must be provided to support them (e.g. planting shrubs, providing suitable safe habitat), (iii) feral animals (e.g. cats, foxes) must be controlled and (iv) the foregone costs of not using the land for more productive purposes. Additionally, many soil-disturbing animals also exert strong trophic-level effects on ecosystems and may compete with livestock. Experimental comparisons of the effects of mechanical and ecological structures on ecosystem functions are needed to promote sustainable and successful reintroduction (table 1).

Given both their trophic and non-trophic effects, we would expect that high densities of soil-disturbing animals would degrade ecosystems, particularly in the absence of natural predators, which is typical in the early days of rewilding programmes [36]. We need a greater research effort to determine appropriate densities of soil-disturbing animals to maximize ecosystem benefits. Very high animal densities may explain, for example, the contrasting results between the beneficial effects reported for pikas and the overall negative effects on ecosystem structure and composition across studies (electronic supplementary material, figure S2). Determining these optimal densities could result in more successful rewilding projects using soil disturbance animals and improve stakeholder acceptance (table 1).

The literature on rewilding with megafauna can help to improve our understanding of the usefulness of soil-disturbing vertebrates for restoration. For example, the tools developed to establish potential reference states (i.e. target composition of animals, [38]) for megafauna may also be useful for soil-disturbing animals too. As in the case of megafauna, it is important to understand how compositional changes in soil-disturbing vertebrates can modulate their effects on ecosystems, and how this might change under future environmental scenarios. For example, the presence of additional species may be necessary to trigger the activity of the soil-disturbing vertebrates (e.g. Figure 2*b*), and thus reintroduction of the latter will be ineffective without the former.

Considering the co-occurrence of several types of soil-disturbing animals in most regions across the globe (figure 1*a*), and their contrasting effects on ecosystem attributes (figure 1*b*; electronic supplementary material, figure S2), it is reasonable to consider the potential for ‘complementarity diversity effects’ (*sensu* [39]; table 1). There will also likely be considerable synergies in promoting a mixed suite of soil-disturbing vertebrates. This could promote a range of different disturbance types and configurations, providing niches for a varied range of plant and animal species. For example, studies in eastern Australia have shown that the capacity to provide safe sites for seeds varies among different soil-disturbing vertebrates [40]. There may also be some redundancy in the ecosystem responses to different soil-disturbing vertebrates given that their populations are likely to oscillate through time in response to changing environmental conditions. Thus, it is likely that diverse assemblages of soil-disturbing animals also show ‘biodiversity portfolio effects’, in which different animals provide the same functional role, but at different times or places [41]. Controlled experiments to test when these biodiversity mechanisms modulate rewilding effects are easier with smaller animals, yet some of the knowledge gathered may be a useful starting point for more targeted evaluations in megafaunal assemblages.

## 6. Concluding remarks

Soil-disturbing vertebrates are diverse and widely distributed across the planet. Their former higher densities, together with substantial evidence on their generally positive effects on ecosystems (particularly on biodiversity and soil fertility) suggest that rewilding with these animals offers a unique opportunity for cheaper and more enduring ecological restoration, with benefits exceeding those using mechanical approaches. However, these potential benefits may depend on the composition of species used and whether high densities can be avoided in the absence of natural predators. There are also potential conflicts with alternative land uses (pastoral or agricultural production) and disservices that need to be accounted for in order to reduce conflict resulting from rewilding. Substantial opportunity exists to explore reference compositions and densities as targets for these rewilding programmes, and to better understand and predict their effects based upon their functional traits and the prevailing environmental and management conditions.

## Data Availability

Data supporting the results have been deposited in the Dryad Digital Repository: https://dx.doi.org/10.5061/dryad.9ghx3ffn8 [42]. The data are provided in the electronic supplementary material [43].
